# Variation in follow-up for children born very preterm in Europe

**DOI:** 10.1093/eurpub/ckad192

**Published:** 2023-11-17

**Authors:** Anna-Veera Seppänen, Henrique Barros, Elizabeth S Draper, Stavros Petrou, Lazaros Andronis, Sungwook Kim, Rolf F Maier, Pernille Pedersen, Janusz Gadzinowski, Véronique Pierrat, Iemke Sarrechia, Jo Lebeer, Ulrika Ådén, Liis Toome, Nicole Thiele, Arno van Heijst, Marina Cuttini, Jennifer Zeitlin, The SHIPS Research Group, J Lebeer, I Sarrechia, P Van Reempts, E Bruneel, E Cloet, A Oostra, E Ortibus, K Boerch, P Pedersen, L Toome, H Varendi, M Männamaa, P Y Ancel, A Burguet, P H Jarreau, V Pierrat, A Nuytten, R F Maier, M Zemlin, B Misselwitz, L Wohlers, M Cuttini, I Croci, V Carnielli, G Ancora, G Faldella, F Ferrari, A van Heijst, C Koopman-Esseboom, J Gadzinowski, J Mazela, A Montgomery, T Pikuła, H Barros, R Costa, C Rodrigues, U Aden, E S Draper, A Fenton, S J Johnson, S Mader, N Thiele, S Petrou, S W Kim, L Andronis, J Zeitlin, A M Aubert, C Bonnet, R El Rafei, A V Seppanen

**Affiliations:** Université de Paris Cité, Inserm, INRAE, Centre for Research in Epidemiology and StatisticS (CRESS), Obstetrical Perinatal and Paediatric Epidemiology Research Team (EPOPé), Paris, France; EPIUnit-Instituto de Saúde Pública da Universidade do Porto, Porto, Portugal; Department of Health Sciences, University of Leicester, Leicester, UK; Nuffield Department of Primary Care Health Sciences, University of Oxford, Oxford, UK; Division of Clinical Trials, Warwick Medical School, University of Warwick, Coventry, UK; Nuffield Department of Primary Care Health Sciences, University of Oxford, Oxford, UK; Children’s Hospital, University Hospital, Philipps University Marburg, Marburg, Germany; Department of Neonatology, Hvidovre Hospital, Hvidovre, Denmark; Department of Neonatology, Poznan University of Medical Sciences, Poznan, Poland; Université de Paris Cité, Inserm, INRAE, Centre for Research in Epidemiology and StatisticS (CRESS), Obstetrical Perinatal and Paediatric Epidemiology Research Team (EPOPé), Paris, France; Department of Family Medicine & Population Health, Faculty of Medicine & Health Sciences, University of Antwerp, Antwerp, Belgium; Department of Family Medicine & Population Health, Faculty of Medicine & Health Sciences, University of Antwerp, Antwerp, Belgium; Department of Women’s and Children’s Health, Karolinska Institutet, Stockholm, Sweden; Department of Neonatal Medicine, Karolinska University Hospital, Stockholm, Sweden; Department of Neonatal and Infant Medicine, Tallinn Children's Hospital, Tallinn, Estonia; Department of Paediatrics, University of Tartu, Tartu, Estonia; European Foundation for the Care of Newborn Infants (EFCNI), Munich, Germany; Department of Neonatology, Radboud University Medical Center, Nijmegen, The Netherlands; Department of Neonatology, Erasmus MC—Sophia Children’s Hospital, Rotterdam, The Netherlands; Clinical Care and Management Innovation Research Area, Bambino Gesù Children’s Hospital, IRCCS, Rome, Italy; Université de Paris Cité, Inserm, INRAE, Centre for Research in Epidemiology and StatisticS (CRESS), Obstetrical Perinatal and Paediatric Epidemiology Research Team (EPOPé), Paris, France

## Abstract

**Background:**

Children born very preterm (<32 weeks of gestation) face high risks of neurodevelopmental and health difficulties compared with children born at term. Follow-up after discharge from the neonatal intensive care unit is essential to ensure early detection and intervention, but data on policy approaches are sparse.

**Methods:**

We investigated the characteristics of follow-up policy and programmes in 11 European countries from 2011 to 2022 using healthcare informant questionnaires and the published/grey literature. We further explored how one aspect of follow-up, its recommended duration, may be reflected in the percent of parents reporting that their children are receiving follow-up services at 5 years of age in these countries using data from an area-based cohort of very preterm births in 2011/12 (*N* = 3635).

**Results:**

Between 2011/12 and 22, the number of countries with follow-up policies or programmes increased from 6 to 11. The policies and programmes were heterogeneous in eligibility criteria, duration and content. In countries that recommended longer follow-up, parent-reported follow-up rates at 5 years of age were higher, especially among the highest risk children, born <28 weeks’ gestation or with birthweight <1000 g: between 42.1% and 70.1%, vs. <20% in most countries without recommendations.

**Conclusions:**

Large variations exist in follow-up policies and programmes for children born very preterm in Europe; differences in recommended duration translate into cross-country disparities in reported follow-up at 5 years of age.

## Introduction

Infants born very preterm (VPT, <32 weeks’ gestation) face an increased risk of health and developmental problems, such as sensory and motor impairments including cerebral palsy, and cognitive and language delay, compared with term-born children.^1–4^ With increasing survival after VPT birth, a growing number of children also present with mild and moderate impairments which can affect their quality of life, school readiness and academic and social skills.^1,2^^,^^5–7^

Because each individual VPT infant’s prognosis is unknown at neonatal discharge, post-discharge follow-up programmes, often managed by neonatal units or follow-up networks for high-risk infants, play a key role in identifying and managing emerging health and developmental problems. They assess children across multiple domains to enable timely and coordinated care.^8^ They also aim to inform and guide families, assess how their child is developing compared with their peers without significant medical history, prepare children for school entry, gain knowledge on long-term outcomes, and provide data for benchmarking.^9^ Follow-up can reduce intensive care hospitalizations and risks of life-threatening illness during the first year of life without increasing costs.^10^ Follow-up is also associated with fewer emergency and sick visits,^11^ possibly by providing better care coordination—identified as an area needing improvement by studies on children with complex care needs,^12^ and by parents of children born VPT.^13^ Follow-up facilitates access to early interventions^14^ which, in turn, can improve motor development in infancy, possibly long-term cognitive outcomes^15^ and parental wellbeing.^16^

Over the past years, follow-up has gained increasing policy priority; national recommendations have recently been issued in the UK by the National Institute for Health and Care Excellence (NICE) (2017),^17^ in France by the National Health Authority (HAS) (2020),^18^ in Denmark by the Danish Paediatric Society (2022)^19^ and in the European Standards of Care for Newborn Health (2018), where follow-up is recommended until, and for some domains beyond, school age.^8^ Nevertheless, no international consensus exists on how these programmes should be organized in terms of eligibility criteria, content or duration. In the EPICE cohort of VPT births from 11 European countries, we found that most VPT children were followed up until 2 years of age, but that wide disparities existed in follow-up between countries at 5 years.^20^

This study investigates follow-up policy and programmes for children born VPT in 11 European countries and explores how policy is reflected in practice by assessing the association between recommendations on follow-up duration and parent reports about whether their children are followed up at 5 years of age.

## Methods

### Study population

The data were collected as part of the Screening to improve Health In very Preterm infantS in Europe (SHIPS) study, a 5-year follow-up of the population-based EPICE cohort of births before 32 weeks’ gestation in 19 regions from 11 European countries: Belgium (Flanders), Denmark (Eastern Region), Estonia (entire country), France (Burgundy, Ile-de-France, the Northern region), Germany (Hesse, Saarland), Italy (Emilia-Romagna, Lazio, Marche), the Netherlands (Central and Eastern region), Poland (Wielkopolska), Portugal (Lisbon, Northern region), Sweden (greater Stockholm) and the UK (East Midlands, Northern, Yorkshire and the Humber regions).^21^

The cohort included all births in these regions over a 12-month or 6-month period (France only) in 2011/12. Out of 7900 live births, 6792 (86.0%) survived to discharge home and 6759 to age 5, of whom 3635 (53.8%) participated in the study at 5 years.

### Data

#### Follow-up policies and programmes

Information was collected on national and regional follow-up policies (recommendations and guidelines) and practices (programmes) in the study regions at the start of the SHIPS follow-up at 5 years. Questionnaires, completed by regional teams in consultation with key informants involved in follow-up programmes, were developed based on a scoping review of the scientific literature on follow-up programmes for children born VPT in Europe. The questionnaires assessed standardized programmes, prioritizing national (first), regional (second) and local (third) programmes and policies, depending on what was available in the country, although limited data were collected on local programmes. Each questionnaire included a question on the availability of programmes: ‘*Do you have national (regional/local) follow-up screening and prevention programmes (or recommendations/guidelines/regulations) for very preterm infants in your country (or region)?’* as well as questions on the year of implementation or issue, screening ages, duration, eligibility criteria, professionals involved, health/developmental areas assessed and assessment methods. With the assistance of country investigators, online sources and grey literature, results from this survey were augmented to describe policies and programmes until March 2022 and those at the cohort’s time of discharge in 2011/12.

#### Perinatal and sociodemographic data

Perinatal and sociodemographic data were abstracted from obstetric and neonatal records, including: gestational age (GA), birthweight (BW), small for GA (SGA) defined as BW < 10th percentile, sex, multiplicity (singleton or one surviving multiple, multiples), bronchopulmonary dysplasia (BPD) defined as supplemental oxygen and/or respiratory support at 36 weeks’ postmenstrual age, intraventricular haemorrhage (IVH) grades III–IV, cystic periventricular leukomalacia (cPVL), retinopathy of prematurity (ROP) stages III–V, necrotizing enterocolitis (NEC) requiring surgery, congenital anomaly and mother’s age and parity at delivery.

#### Parent’s report of children’s use of follow-up services at 5 years of age

Information on use of follow-up services came from a question in the parental questionnaires when the children were 5 years of age: ‘*Does your child have routine check-ups for children who were born prematurely [optional description of local/regional service]? No, never had such check-ups; No, not anymore (please specify age at last check-up); Yes, still has check-ups (at neonatal unit where he or she was born, at other place or healthcare professional…)’.* The question was translated and adapted for each country (Supplementary table S1). Additional sociodemographic characteristics were collected at 5 years on maternal educational level and country of birth (native, non-native European-born, or born outside Europe).

### Analysis

We first summarized information on national and regional follow-up policies and programmes with priority given to describing national recommendations and guidelines over programmes, as there may be variation in programme implementation. For each country, we described the proportions of parents reporting continued use of follow-up services for children born VPT at 5 years of age. To account for differences in population characteristics, case-mix adjusted proportions were generated using predicted margins from logistic regressions with robust variance estimators for clustering within countries. This model adjusted for the sociodemographic and perinatal characteristics described above.

We created risk group classifications based on criteria associated with poor long-term outcomes. The principal classification was based on GA and BW (<28 weeks and/or <1000 g; 28–29 weeks and ≥1000 g; 30–31 weeks and ≥1000 g). A second classification included the presence of at least one neonatal risk factor (GA < 28 weeks and/or BW < 1000 g; GA ≥ 28 weeks and BW ≥ 1000 g with BPD, severe congenital anomaly, IVH grades III–IV, cPVL, ROP stages III–V or NEC requiring surgery; GA ≥ 28 weeks and BW ≥ 1000 g without neonatal risk factors). To take into consideration potential bias due to attrition,^21^ inverse probability weights after multiple imputation with chained equations were used for all analyses, giving a higher weight to children with characteristics of non-responders. STATA 14.2 was used for all analyses (Stata Corp., College Station, TX, USA).

### Ethics

All study regions obtained ethics approvals from local committees and informed consent from parents for follow-up according to national legislation. The SHIPS study was approved by the French Advisory Committee on Use of Health Data in Medical Research (CCTIRS) and the French National Commission for Data Protection and Liberties (CNIL).

## Results

In 2011/12, national or regional programmes or policies were established in 6 of 11 countries, and by 2015/16 in all but two countries: Denmark and the UK (table 1). National guidelines were published for the UK in 2017 by NICE^17^ and by the Danish Paediatric Community in 2022.^19^ Three countries had established follow-up programmes without preceding national recommendations: Belgium and Sweden with national-level programmes, and Denmark with local programmes only, until the national guidelines were published in 2022. The eligibility criteria of the most recent follow-up programmes are based on GA and/or BW (all countries) and intrauterine growth restriction (IUGR) status (Estonia, France and Sweden). Most countries recommend follow-up of all infants born <32 weeks’ gestation or <1500 g, whereas the UK recommends follow-up for all infants born <30 weeks, and Sweden for those born <28 weeks or with a BW <−3 SD. Other perinatal risk factors, such as severe neonatal morbidities, morphological brain damage, severe asphyxia and cardiopathies regardless of GA, defined additional eligibility in Belgium, Estonia, France, Italy, the Netherlands, Sweden and the UK. In 2011/12, three countries (France, the Netherlands and Portugal) had a national or regional policy that recommended follow-up of all children born <32 weeks’ GA or <1500 g until at least 5 years of age. Two additional countries did so from 2015/16 onwards: Belgium (for all children born <32 weeks’ GA or <1500 g) and Sweden (for children born <28 weeks’ GA or with a BW <−3 SD). In 2022, Denmark also recommended follow-up for children born <28 weeks’ GA until 5 years.

**Table 1:** Eligibility criteria and follow-up duration of national or regional follow-up policies or programmes

	2011/12		2015/16		March 2022	
	*n*	Country	*n*	Country	*n*	Country
Local programmes only	5	BE, DK, ITA, SE, UK	2	DK, UK	0	
National or regional recommendation^a^ or programme (year of issue/last update)	6	DE (2010),^b^ EE (2008),^b^ FR (2010–11),^c^ NL (2012),^b^ PL (1998),^c^ PT (2012)^b^	9	BE (2014),^d^ DE (2014),^b^ EE (2008),^b^ FR (2014),^c^ ITA (2015),^b^ NL (2015),^b^ PL (2015),^b^ PT (2012),^b^ SE (2015)^d^	11	BE (2014),^d^ DE (2020),^b^ DK (2022),^b^ EE (2008),^b^ FR (2020),^b^ ITA (2015),^b^ NL (2015),^b^ PL (2015),^b^ PT (2012),^b^ SE (2015),^d^ UK (2017)^b^
**Eligibility criteria of national or regional recommendations or programmes (multiple options possible)**						
Routinely for all babies born <32 WG or <1500 g	6	DE, EE, FR, NL,^e^ PL, PT	8	BE, DE, EE, FR, ITA, NL,^e^ PL, PT	9	BE, DE, DK, EE, FR, ITA, NL,^e^ PL, PT
Routinely for all babies born <30 WG	6	DE, EE, FR, NL,^e^ PL, PT	8	BE, DE, EE, FR, ITA, NL,^e^ PL, PT	10	BE, DE, DK, EE, FR, ITA, NL,^e^ PL, PT, UK
Routinely for all babies born <28 WG or <1000 g	6	DE, EE, FR, NL,^e^ PL, PT	9	BE, DE, EE, FR, ITA, NL,^e^ PL, PT, SE	11	BE, DE, DK, EE, FR, ITA, NL,^e^ PL, PT, SE, UK
Routinely for all babies born <37 WG with IUGR (EE, FR <3rd percentile) or BW< −3 SD (SE)	2	EE, FR	3	EE, FR, SE	3	EE, FR, SE
Routinely for all babies with other perinatal risk factors regardless of GA^f^	3	EE, FR, NL	6	BE, EE, FR, ITA, NL, SE	7	BE, EE, FR, ITA, NL, SE, UK
**Follow-up duration, until years of age** ^g^						
Recommended follow-up or programme until 2 only	3	DE, EE, PL	2	DE, EE	2	DE, EE
Recommended follow-up or programme until 3 or 4 years only	0		2	ITA, PL	3	ITA, PL, UK^h^
Recommended follow-up or programme until 5 years of age or longer	3	FR,^c^ PT, NL	5	BE, FR, NL, PT, SE	6	BE, DK,^i^ FR, NL, PT, SE

a Formalized in official document.

b National recommendation or guideline; regional and local programmes may differ in practice.

c Regional: regional programme in Poland (Wielkopolska); semi-regional network policies in France (Ile-de-France—eligibility and duration as defined in 2015 may differ across networks in 2011/2012).

d National programme.

e BW < 1500 g and <10 pctle.

f BE: Asphyxia, congenital cardiopathy, other congenital and/or chromosomal disorders, abnormalities on clinical neurological examination at discharge from NICU and/or brain ultrasound, congenital infections or hyperbilirubinemia; EE: severe congenital anomalies, metabolic disorders, morphological brain damage, severe asphyxia or severe encephalopathy, cerebral infections, hyperbilirubinemia above the level of exchange transfusion, failure to thrive in the NICU, maternal substance abuse and different pathologies at the decision of the neonatologist; FR: other adverse perinatal outcomes such as congenital anomalies or cardiopathies; ITA: morbidities such as asphyxia and genetic diseases; NL: brain damage or abnormalities, asphyxia or treatment for hypothermia, and infants referred to top referral care, for instance when born after foetal therapy; SE: Morphological brain damage, severe asphyxia or severe encephalopathy, cerebral infections or other severe morbidity; UK: Other perinatal risk factors such as brain lesions or asphyxia.

g If follow-up duration differs for subgroups of children, longest follow-up is given.

h Four years if GA <28 weeks, 2 years if GA 28–29 weeks.

i Five years if GA <28 weeks, 2 years if GA 28–31 weeks.

BE, Belgium; BW, birth weight; DE, Germany; DK, Denmark; EE, Estonia; FR, France; GA, gestational age; ITA, Italy; IUGR, intrauterine growth restriction; NL, the Netherlands; PL, Poland; PT, Portugal; SD, standard deviations; SE, Sweden; UK, the United Kingdom; WG, weeks’ gestation.

The most recent policies and programmes include between one (Germany) and 10–12 (Poland) assessment visits at varying time points (table 2). Policies prescribe which assessments and examinations should be performed, except in Portugal, where this is decided in the neonatal units. All policies or programmes recommend neurodevelopmental assessments, but vary for other assessments, such as vision and hearing, socio-behavioural, speech and growth evaluations. Professionals involved in the follow-up include neonatologists, paediatricians, clinical psychologists, physiotherapists or other paediatric subspecialties, such as child neurologists and speech therapists.

**Table 2:** Characteristics of most recent follow-up policies and programmes

Country	Policy or programme (year of issue or update)	Assessment frequency	Assessments performed	Professionals involved
Belgium	National programme (2014) by RIZIV—INAMI	Four assessments: 3–5 months’ CA (A); 9–13 months’ CA (B); 22–25 months’ CA (C); 4.5–5.5 years (D)	A: General paediatric evaluation, neurological exam, general movement, growth, evaluation of sensory development, parenting (+EPDS) and neuro-motor evaluation (fine and gross motor skills) B: A + mental examination (cognition and communication) C: B + prosocial behaviour D: C + behaviour, language, preschool skills/spatial awareness, writing skills	Neonatologist, neurologist, clinical psychologist, speech therapist, physiotherapist, social worker, in cooperation with paediatrician, social network etc. in collaboration with Centres for Developmental Disabilities
Denmark	National guideline (2022) by the Danish Paediatric Society	Four to six assessments: 3–5 months’ CA; 5–12 months’ CA; 18 months’ CA; 24 months’ CA; 36 months’ CA (GA < 28 weeks only); 60 months’ CA (GA < 28 weeks only)	Neurological assessment, assessments of growth, nutrition and infections, special needs and worries by parents at each visit, developmental assessment (ASQ) at 24 months	Neonatologist, physiotherapist, ergotherapist, ophthalmologist and paediatric neurologist or pulmonologist in the case of neurological or respiratory symptoms at follow-up
Estonia	National recommendation (2008) supported by the Estonian Health Insurance Fund	Six assessments: 2 months’ CA; 4 months’ CA; 6 months’ CA; 12 months’ CA; 18 months’ CA; 24 months’ CA	Paediatrician at neonatal unit, physiotherapist (2, 4, 6, 9 and 12 months) Otoacoustic emissions, brainstem auditory evoked potential (9 months only) Vision test (12 months) Physiotherapist assessment (18 months) Developmental and speech assessments (24 months) According to individual need: child neurologist, other paediatric subspecialists	Paediatricians, clinical psychologists, physiotherapists, child neurologist and other paediatric subspecialists
France	National guideline (2020) by the HAS	Six assessments: 9 months’ CA; 18 months’ CA; 24 months’ CA; 30–36 months; 4 years; 5 years	Motor, language, social and behavioural development, working memory, learning abilities, hearing, vision and growth assessments	Generalists, paediatricians, staff of medico-social action centres and general maternal and child follow-up services (PMI, CAMSP, CMP, CMPP), nurses, social workers, physiotherapists, psychomotor therapists, speech therapists, orthoptists, ergotherapists, school nurses, paediatric psychiatrists, psychologists, neuro-paediatricians
Germany	National guideline (2020) (QFR-RL) by the Gemeinsamer Bundesausschuss Institution (G-BA)	One assessment: 24 months’ CA	Neuro-motor development, cognition, hearing, vision. Standardized developmental test (Bayley III) recommended	Responsible for follow-up: neonatal unit. Most commonly performed in the neonatal unit, the department of neuropaediatrics or the social paediatric centre by paediatricians, developmental psychologists and physiotherapists
Italy	National recommendation (2015) by the Italian Society of Neonatology	Seven assessments: 7–10 days after discharge; 40 weeks’ CA; 2–3 months’ CA; 6–8 months’ CA; 12–14 months’ CA; 18–24 months’ CA; 36 months’ CA	Neuromotor development, growth and nutrition, vision, hearing, communication and language skills, respiratory function and quality of life	A neonatologist or paediatrician as follow-up coordinator, a paediatric neurologist, a psychologist, a physiotherapist or neuropsychomotor specialist, a nurse and a secretary (administrative staff) The following professionals should be available for consultation (not part of the team): ophthalmologist or eye specialist, hearing specialist, paediatric cardiologist, paediatric neurosurgeon and orthopaedic
The Netherlands	National guideline (2015) by the national working group on neonatal follow-up	Five assessments: 6 months’ CA; 12 months’ CA; 2 years’ CA; 5 years’ CA; 8 years’ CA	Background data, physical, neurological, motor examination (6 and 12 months) Anamnesis, paediatric, neurological, IQ, speech, language, visual-cognitive, motor and behavioural examinations (covered in examinations at 2, 5, 8 years)	Paediatricians, developmental psychologists, speech and physiotherapists
Poland	National recommendation (2015) by the Polish Neonatal Society	Ten to twelve assessments: 1 month; 3 months; 6 months; 9 months; 12 months; second year: 2–4 visits (compulsory at 18 and 24 months); third year: three visits	Postnatal growth, physical examination, laboratory tests, assessment of endocrinology activity, psychomotor development with standardized tests, eye examination and if needed: ENT, cardiology, speech	Regional Perinatal Centres, including paediatrician, developmental psychologist, physiotherapist, speech therapist and health visitor
Portugal	National recommendation (2012) by the Neonatology Section of the Portuguese Society of Paediatrics	Eight assessments: 40 weeks’ CA to 1 months’ CA; 6 months’ CA; 12 months’ CA; 18–24 months’ CA; 30 months’ CA; 3–4 years; 5–6 years; 8 years	Non-standardized, unit-based, routine follow-up: Hearing, vision, neurological, psychomotor, growth, mental development, gross motor, school performance assessments, with more specific assessments if dysfunctions (feeding, behaviour, autism, IQ, language and dyslexia)	Clinicians at unit of hospitalization (neonatologist, development paediatrician, psychologist, ORL, ophthalmologist, physiotherapist) or local hospital/health care centre (GP, specialists according to need, early childhood intervention programmes only for children specially identified and referred)
Sweden	National programme (2015), by the National Neonatal Association	Five to six assessments: Minimum 3–4 visits before 2 years’ CA; 2 years’ CA; 5.5 years	Neurocognitive, developmental, motor, behavioural and growth assessments at 2 years’ CA, with added mental health, lung and blood pressure assessments at 5 years	Paediatrician, neonatologist, developmental psychologist and speech therapist
UK	National guideline (2017) by NICE	Three assessments: 3–5 months’ CA; 12 months’ CA; 24 months’ CA Additional developmental assessment at 4 years if GA < 28 weeks	In-depth assessment at 24 months includes development, motor, attention, emotional, behavioural, vision, hearing, feeding, sleeping and growth assessments	Multidisciplinary team with expertise in neonatal care, development of children born very preterm, providing support in the community, administering and interpreting results from questionnaires and standardized tests, collating information for decision-making and writing reports, and local care pathways

RIZIV, Rijksinstituut voor Ziekte-en Invaliditeitsverzekering; INAMI, Institut national d'assurance maladie-invalidité; CA, corrected age; EPDS, Edinburgh Postnatal Depression Scale; GA, gestational age; BW, birth weight; ASQ, ages and stages questionnaire; HAS, Haute Autorité de Santé; PMI, Protection Maternelle et Infantile; CAMSP, Centre d'action médico-sociale précoce; CMP, Centre médico-psychologique; CMPP, Centre médico-psycho-pédagogique; QFR-RL, Qualitätssicherungs-Richtlinie Früh- und Reifgeborene; IQ, intelligence quotient; ORL, Otorhinolaryngologist; GP, general practitioner; NICE, National Institute for Health and Care Excellence.

In the study sample, mean GA was 28.8 [standard deviation (SD): 2.8] weeks and BW was 1241 (SD: 369) grams. Over one-quarter of children were born before 28 weeks’ GA, while 13.3% had BPD and 10.3% severe non-respiratory morbidity (table 3). The clinical characteristics of responders and non-responders were generally similar, although responders had slightly lower GA with fewer younger, foreign-born and multiparous mothers (table 3).

**Table 3:** Perinatal and social characteristics of responders and non-responders

	Responders at 5 years		Non-responders at 5 years		
	*N* = 3635		*N* = 3124		
	*n*	%	*n*	%	*P*
Gestational age, completed weeks					
<26	306	8.4	236	7.6	<0.001
26–27	663	18.2	449	14.4	
28–29	945	26	878	28.1	
30–31	1721	47.4	1561	50	
Missing	(0)	(0)	(0)	(0)	
Small for gestational age (percentiles)					
<3rd	776	21.4	613	19.6	0.199
3–9th	419	11.5	378	12.1	
≥10th	2440	67.1	2133	68.3	
Missing	(0)	(0)	(0)	(0)	
Congenital anomalies					
No	3336	91.8	2872	91.9	0.812
Yes	299	8.2	252	8.1	
Missing	(0)	(0)	(0)	(0)	
Bronchopulmonary dysplasia					
No	3077	86.8	2600	85.1	0.059
Yes	470	13.3	454	14.9	
Missing	(88)	(2.4)	(70)	(2.2)	
Severe non-respiratory morbidity^a^					
No	3186	89.7	2690	89.2	0.528
Yes	367	10.3	326	10.8	
Missing	(82)	(2.3)	(108	(3.5)	
Child sex					
Male	1938	53.3	1689	54.1	0.528
Female	1697	46.7	1434	45.9	
Missing	(0)	(0)	(1)	(0.0)	
Multiple birth					
Singleton or one surviving multiple	2567	70.6	2227	71.3	0.52
Multiples (twins, triplets or quadruplets)	1068	29.4	895	28.7	
Missing	(0)	(0)	(2)	(0.1)	
Maternal age at delivery					
≤24 years	428	11.8	695	22.4	<0.001
25–34 years	2084	57.5	1736	55.9	
≥35 years	1113	30.7	677	21.8	
Missing	(10)	(0.3)	(16)	(0.5)	
Parity					
Multiparous	1417	39.4	1635	71.9	<0.001
Nulliparous	2177	60.6	1558	6.3	
Missing	(41)	(1.1)	(69)	(2.2)	
Maternal education^b^^,^^c^					
Lower	599	16.8			
Intermediate	1483	41.5			
Higher	1492	41.8			
Missing	(61)	(1.7)			
Employment status^c^					
No parent unemployed	3161	88.8			
At least one parent unemployed	399	11.2			
Missing	(75)	(2.1)			
Maternal country of birth					
Native	2890	79.9	2115	71.9	<0.001
European-born	243	6.7	184	6.3	
Born outside Europe	486	13.4	643	21.9	
Missing	(16)	(0.4)	(182)	(5.8)	

a Intraventricular haemorrhage grades III–IV, cystic periventricular leukomalacia, retinopathy of prematurity stages III–V or necrotizing enterocolitis requiring surgery.

b Lower: ISCED levels 0–2 (lower secondary); intermediate: ISCED levels 3–5 (upper or post-secondary, non-tertiary or short cycle tertiary); higher: ISCED levels 6–8 (Bachelor’s degree or higher).

c Data items collected at 5 years only.

**Figure 1:**
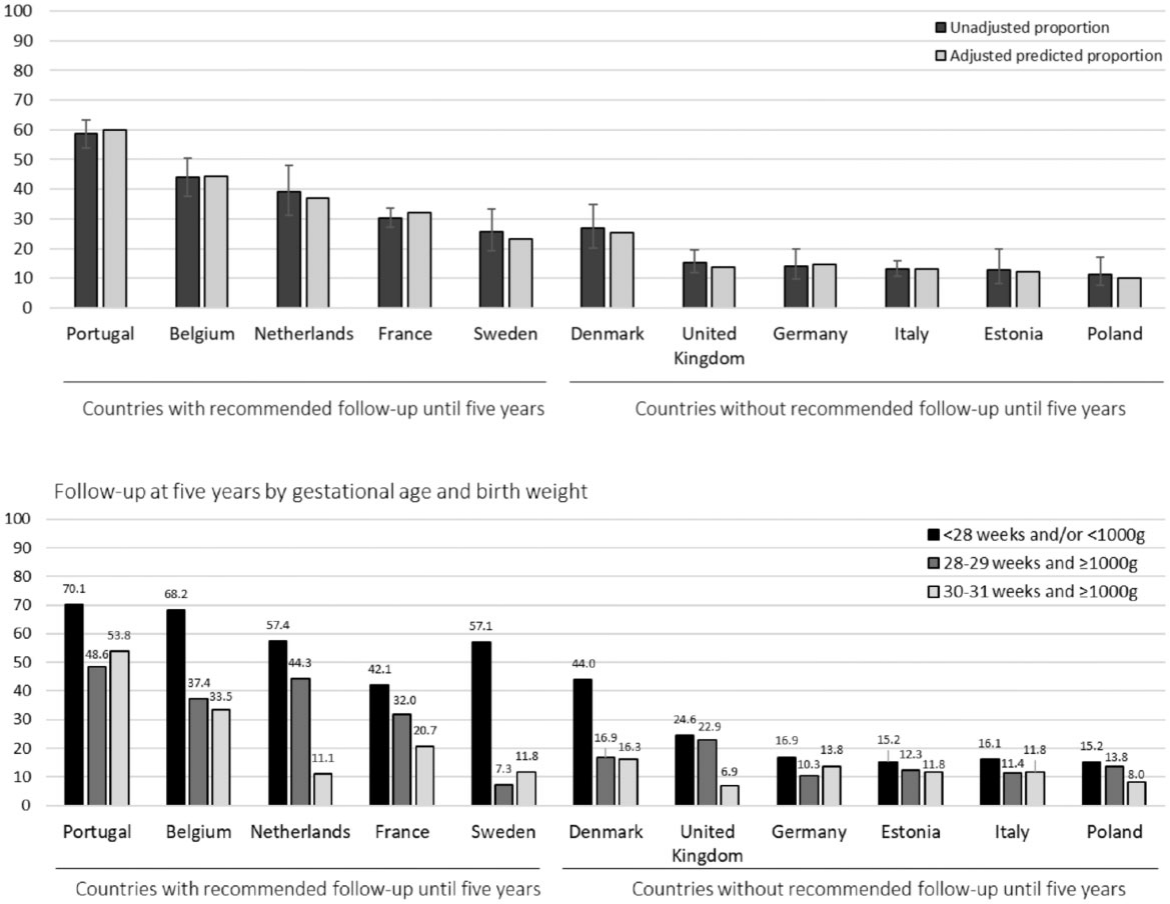
Overall unadjusted and adjusted^a^ proportions^b^ of parent-reported follow-up and reported follow-up by perinatal risk defined by GA and BW for children born <32 weeks’ gestation in follow-up at 5 years of age by country.^c^*Notes:*^a^Adjusted for GA, SGA, BPD, IVH grades III–IV, cPVL, ROP stages III–V, NEC requiring surgery any congenital anomaly, child sex, multiple birth, maternal educational level, maternal age at delivery, maternal country of birth and parity. ^b^All proportions have been estimated using inverse probability weights. ^c^Sample sizes: Portugal (Lisbon and Northern region): 254/425; Belgium (Flanders): 114/259; the Netherlands (Central and Eastern region): 58/146; France (Burgundy, Ile-de-France and the Northern region): 246/770; Sweden (greater Stockholm): 40/141; Denmark (Eastern Region): 43/151; the UK (East Midlands, Northern, Yorkshire and the Humber regions): 64/419; Germany (Hesse and Saarland): 37/266; Italy (Emilia-Romagna, Lazio and Marche): 93/691; Estonia (entire country): 17/133; Poland (Wielkopolska): 21/186.

Figure 1 (upper panel) shows the proportion of parents reporting that their children were still using follow-up services at 5 years of age. This varied between 11.4% (95% CI: 7.5–17.0) in Poland and 58.7% (95% CI: 53.8–63.4) in Portugal. Countries with national or regional recommendations for follow-up until 5 years of age in 2015/16 (Portugal, Belgium, the Netherlands, and France) had the highest proportions of parents reporting continued use of follow-up services (with the exception of Denmark that had similar rates as Sweden). These differences were not explained by clinical and socioeconomic characteristics as shown by minor changes in case-mix adjusted proportions. Figure 1 (lower panel) illustrates parent-reported use of follow-up services by the children’s levels of perinatal risk, as defined by GA and BW. Reported follow-up at 5 years of age was highest in children born <28 weeks’ gestation and/or with a BW of <1000 g, with variation between 42.1% (France) and 70.1% (Portugal) in countries with recommended follow-up until 5 years, and between 15.2% (Poland, Estonia) and 44.0% (Denmark) in countries without such recommendations in 2015/16. The second classification integrating neonatal risk factors (Supplementary figure S1) reported follow-up was highest in the group of children with GA ≥28 weeks and/or BW ≥1000 g with a neonatal risk factor (the Netherlands, the UK, Germany and Poland), but in five countries (Portugal, France, Denmark, Estonia and Italy) it was lower than for children born at ≥28 weeks and/or BW ≥1000 g *without* neonatal risk factors, although the sample size for this group was small in some countries (see notes in Supplementary figure S1).

## Discussion

Our study showed increasing adoption of national follow-up policies for children born VPT in 11 European countries over the last decade, suggesting that their health and development is gaining recognition as a public health priority. However, high variability in the eligibility criteria, recommended duration and content of policies between countries illustrates that consensus is lacking concerning the optimal organization of these programmes. Using one key element of follow-up—its duration—we were able to show differences in population-level reported follow-up service use related to policies at the country level. Cohort data from the SHIPS study showed wide variations in parent-reported use of follow-up services in children at 5 years of age. In particular, reported follow-up of children with perinatal risk factors for future health and developmental problems, commonly used as eligibility criteria to ensure follow-up of these sub-groups, was strikingly lower at 5 years when national or regional follow-up policies were not in place to ensure follow-up beyond 2–4 years.

Strengths of our study include using multi-national European data from countries with universal healthcare coverage and similar living standards to compare follow-up policies and use of follow-up services in subgroups of children with perinatal risk factors. Previous international policy comparisons of policies have successfully provided new knowledge for policy decisions, for instance in the MOCHA study, which showed variation in paediatric care policies, healthcare delivery and quality of care assessments across 30 European countries.^12^^,^^22^ Limitations include that less data were collected on local compared with national or regional programmes, and not being able to fully account for diversity in healthcare systems that affect policy implementation. Further, our data come from selected regions and do not represent practices nationally. We used data from parent-report questionnaires to assess the use of follow-up services; their responses may be affected by their understanding of follow-up services and subject to misinterpretation, although parents’ perception of the follow-up care that their children are receiving is important information as such. Further, studies have shown that children participating in research studies are more likely to participate in neurodevelopmental follow-up,^23^ which may have led to an overestimation of follow-up service use in our analyses among the families that were included in the follow-up. However, our sample was population-based and we used inverse probability weights to take into account non-response. Finally, we used data from a large, population-based study, but numbers of cases in certain subgroups remain small, and results need to be interpreted with caution.

Few reports in the literature investigate or evaluate follow-up programmes for children born VPT which makes it difficult to compare between countries. However, existing studies have suggested that, in the absence of follow-up recommendations, follow-up often ceases at 2 years of age and its application can be highly heterogeneous. In New Zealand, where there are no national guidelines for follow-up, and neonatal intensive care units (NICUs) are responsible for following up their patients, inclusion criteria tend to vary by GA, BW and clinical characteristics, and duration remains at the discretion of the clinician and is therefore often discontinued if developmental delays have not been identified by 2 years of age, mainly due to lack of funding and resources.^24^ Similarly, in Australia, NICUs are responsible for the follow-up of all infants born <32 weeks’ gestation (personal communication, Peter Anderson, Professor of Paediatric Neuropsychology, Monash University). In the USA, a survey in 2012 showed high variability in follow-up programmes for high-risk children partly due to resources being dependent on a neonatal unit’s association with academic centres.^25^ A Spanish study showed that 71% of NICUs in the country offered follow-up for VPT-born infants prior to the publication of national recommendations, but that heterogeneity in programme content was high and no unit fulfilled the recommendations that were later published in 2017.^26^

Our study showed that parents’ reported follow-up service use at 5 years was consistently higher in countries with long-term follow-up policies compared with countries without, but it was not universal even when this was recommended. Further, the variation between countries was highest for children at most risk. This finding contradicts the assumption that high-risk children continue follow-up anyway in the absence of policy recommendations. We also found that children with severe morbidities at higher GA or BW may not be specifically targeted for follow-up, despite the association between risk factors such as BPD or severe brain haemorrhage and poor long-term outcomes. Risk factors such as neonatal morbidities, but also social vulnerabilities, may need more attention in follow-up and interventions to improve outcomes.^27–29^ Neighbourhood-level social deprivation,^30^ family socioeconomic status and demographic characteristics^31^ may be considered for improving timely access to diagnosis and care.^32^

While our study and other studies suggest that policy is important for guiding practice, follow-up requires funding,^33^ resources^9^^,^^25^ and appropriate methods for successful implementation.^34^ We observed high variability in parent-reported follow-up at 5 years even among countries with long-term follow-up policy which may reflect differences in practical implementation; some countries’ national/regional policies are accompanied by national/regional programmes, whereas others are not, and local programmes may be inconsistent with the policies described here. More research is needed to assess healthcare system-related factors that may affect use of follow-up services, including organization of primary care,^35^ gate-keeping systems and hospital or community-based service provision.^36^ Non-use of follow-up services may also stem from poor organization (e.g. failure to inform parents about follow-up)^37^ or individual barriers (e.g. ability to pay for out-of-pocket costs^38^ or to attend appointments^39^). Finally, while follow-up programmes provide screening for developmental and health problems, further evaluation is needed on their ability to improve outcomes, through referral of children to further care,^40^ and the availability of early interventions.

## Conclusions

The organization of follow-up services for children born VPT differs greatly across European, with large variations in parent-reported use of follow-up service use at 5 years of age, even in children facing the greatest risks of health and developmental problems. Although variation is expected, low proportions of parents reporting follow-up service use for their at-risk children may suggest sub-optimal support for high-risk children and their families. While progress is evident in the many countries that have recently implemented or expanded recommendations on follow-up, the marked differences in organizational characteristics and content revealed in our study provide an opportunity to reflect on how they can be improved and on areas to be targeted for further research.

## Supplementary Material

ckad192_Supplementary_Data

## Data Availability

The data underlying this article are available in the article and in its online supplementary material.
