# Supplementary material for: Variation in follow-up for children born very preterm in Europe
Source: Eur J Public Health. 2023 Nov 17;34(1):91–100. doi: 10.1093/eurpub/ckad192 (PMC10843937; doi:10.1093/eurpub/ckad192)
Supplement: ckad192_Supplementary_Data [file ckad192_supplementary_data.zip › ckad192_Supplementary_Data/ejph-2023-08-om-0454-File003.docx]

|  | **Translatio** | **Back-translation** |
| --- | --- | --- |
| Belgium | Heeft uw kind standaardcontroles voor kinderen die te vroeg zijn geboren? (Centrum voor Ontwikkelingsstoornissen, kinderarts)  …Bij de afdeling neonatologie/ kindergeneeskunde waar hij of zij is geboren  …Andere plaats of professionele zorgverlener: | Does your child receive standard check-ups for children who were born prematurely? (Centre for Developmental Disabilities, paediatrician)  ...Neonatology department where he or she was born  ...Other location or the professional healthcare provider: |
| Denmark | Bliver jeres barn fulgt op af rutine – eller opfølgningsundersøgelser for børn født for tidligt?  ….Neonatal afdeling (afdeling for nyfødte) hvor vi var indlagt efter fødslen ….Andre steder og andet sundhedspersonale, angiv hvor og hvilke: | Is your child being followed up by routine or follow-up examinations for children born preterm?  …Neonatal unit (unit for newborn) where we were hospitalized after birth  …Other place or other health care professional, please specify where and which ones: |
| Estonia | Kas Teie laps on käinud enneaegsetele lastele mõeldud rutiinses tervisekontrollis? …Vastsündinute osakond, kus laps sündis …Muu koht või terviseteenuse asutus: | Has your child gone through a routine health check for premature children? …Neonatal unit the child was born in …Other place or health care institution: |
| France | Votre enfant a-t-elle/il des examens de santé dans le cadre d’un suivi spécifique aux enfants nés prématurés ?  … dans le service de néonatologie dans lequel il a été pris en charge après sa naissance … autre, préciser : | Does your child have health examinations as part of a follow-up specifically for children born preterm?  ...in the neonatal service where he was taken care of after birth …other, specify: |
| Germany | Erhält Ihr Kind spezielle Nachsorgeuntersuchungen für frühgeborene Kinder [zusätzlich zu den üblichen U-Untersuchungen]?  …Neugeborenenstation, wo es nach der Geburt betreut wurde  …Sonstige Einrichtung oder medizinische Fachkraft: | Does your child receive special follow up examination for preterm infants [in addition to the common examinations for all children]?  …Neonatal unit, where it was treated after birth  …Other facility or medical professional: |
| Italy | Il suo bambino segue un programma di controlli periodici (“follow-up”) per bambini nati pretermine?  … Unità di Terapia Neonatale/Neonatologia dell’Ospedale di nascita/dimissione  … Altra struttura sanitaria oppure figura sanitaria (ad es. pediatra di base, neuropsichiatra, psicologa…): | Does your child follow a programme of regular check-ups ("follow-up") for children born preterm?  ...NICU/neonatal unit of the hospital of birth or discharge  ...Other institute or healthcare professional (e.g. paediatric GP, paediatric neurologist, psychologist...): |
| The Netherlands | Wordt uw kind nog routinematig opgevolgd vanwege zijn/haar prematuriteit? (bijvoorbeeld in een Centrum voor Ontwikkelingsstoornissen (COS), kinderarts,…)  …Neonatologie waar hij/zij geboren werd  …Andere plaats of hulpverlener: | Does your child receive standard check-ups for children who were born prematurely? (Outpatient Follow-up Clinic)  … Neonatology department where he or she was born  …Other location or other healthcare provider: |
| Poland | Czy dziecko przechodzi rutynowe badania kontrolne dla dzieci urodzonych przedwcześnie?  …Oddział neonatologii, na którym dziecko przebywało po urodzeniu  …Inna placówka lub pracownik służby zdrowia | Does your child have routine check-ups for preterm children?  … Neonatology ward where the child stayed after birth  …Another facility or health care professional |
| Portugal | O/a seu/sua filho/a faz consultas de rotina específicas para crianças nascidas prematuramente?  …Unidade de cuidados neonatais onde a criança nasceu  …Outro local ou profissional de saúde: | Does your son/daughter have routine appointments specifically for prematurely born children?  …Neonatal care unit where the child was born  ...Other location or healthcare professional: |
| Sweden | Följer ditt barn på ett uppföljningsprogram för prematurfödda, t.ex vid neonatalmottagning?  …Neonatal vårdenhet/mottagning där barnet vårdades som nyfödd  … Annan vårdinrättning eller vårdkontakt: | Is your child in a follow-up programme for children born preterm, e.g. at a neonatal clinic?  …Neonatal care unit/ facility where the child was cared for as newborn  …Other healthcare facility or healthcare contact: |
| The United Kingdom | Does your child have routine check-ups for children who were born prematurely? …In neonatal unit where he or she was born …other place or health care professional: | |

**eTable 1:** **Translations and back-translations for questions on follow-up by country**
