# Supplementary material for: Variation in follow-up for children born very preterm in Europe
Source: Eur J Public Health. 2023 Nov 17;34(1):91–100. doi: 10.1093/eurpub/ckad192 (PMC10843937; doi:10.1093/eurpub/ckad192)
Supplement: ckad192_Supplementary_Data [file ckad192_supplementary_data.zip › ckad192_Supplementary_Data/ejph-2023-08-om-0454-File004.docx]

**Figure S1**. Estimated proportions^a^ of parent-reported follow-up for children at five years of age by country, gestational age, birth weight and perinatal risk^b,c^

^
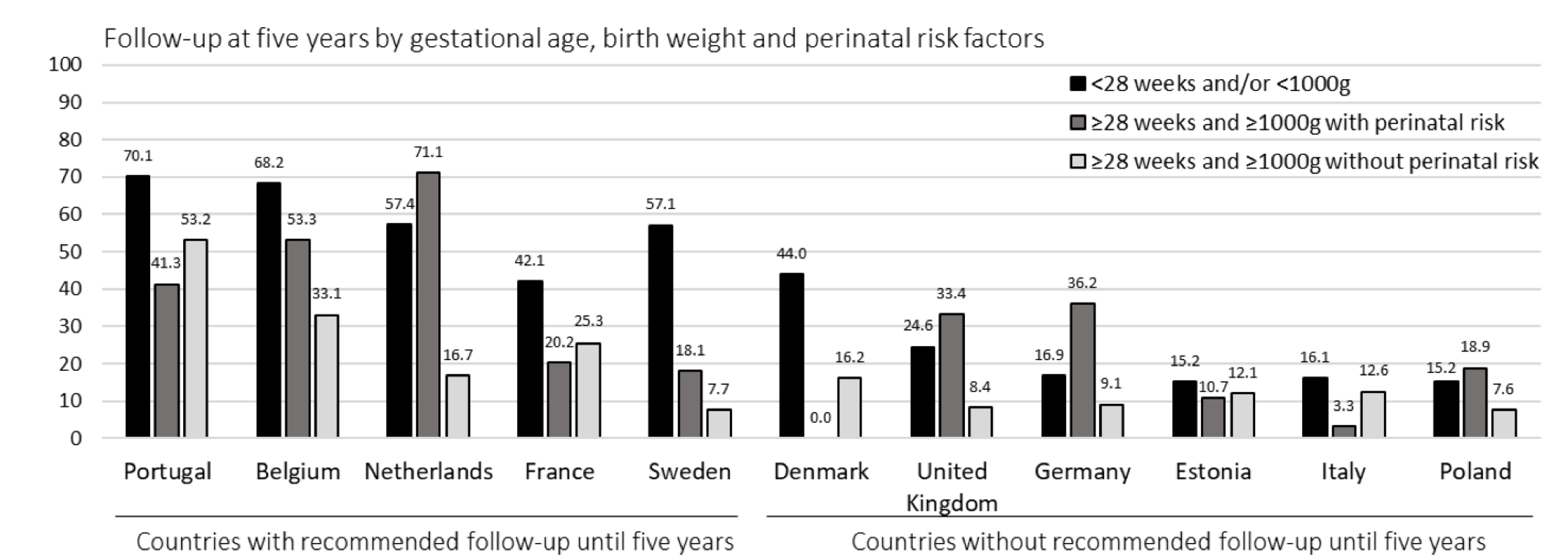
^

Notes: ^a^All proportions have been estimated using inverse probability weights. ^b^Bronchopulmonary dysplasia, severe congenital anomaly, intraventricular haemorrhage grades III–IV, cystic periventricular leukomalacia, retinopathy of prematurity stages III–V or necrotizing enterocolitis requiring surgery. ^c^Sample sizes (n children followed up at five years in subgroup/N children in subgroup) ≥28 weeks and ≥1000g with perinatal risk: Portugal: 9/21; Belgium: 6/12; Netherlands: 3/5; France: 8/36; Denmark: 1/6; Sweden: 0/6; United Kingdom: 12/42; Germany: 4/17; Estonia: 1/10; Italy: 1/31; Poland: 4/20; ≥28 weeks and ≥1000g without perinatal risk: Portugal: 131/242; Belgium: 50/152; Netherlands: 12/63; France: 121/453; Denmark: 7/71; Sweden: 11/79; United Kingdom: 19/226; Germany: 15/147; Estonia: 10/83; Italy: 53/413; Poland: 7/102.
